# Fatal toxoplasmosis in a captive squirrel monkey (*Saimiri boliviensis*) in Portugal

**DOI:** 10.1007/s11259-023-10179-x

**Published:** 2023-07-21

**Authors:** Martha Ynés Salas-Fajardo, Julio Benavides, Alexandre Azevedo, Paulo Figueiras, Madalena Monteiro, Leonor Orge, Paula Mendonça, Paulo Carvalho, Helga Waap, Luis Miguel Ortega-Mora, Rafael Calero-Bernal

**Affiliations:** 1 SALUVET, Department of Animal Health, Faculty of Veterinary, Complutense University of Madrid, Madrid, 28040 Spain; 2 Instituto de Ganadería de Montaña, (CSIC-ULE), Grulleros, León, 24346 Spain; 3 Zoo de Lagos, Lagos, 8600-013 Portugal; 4 CIVG – Vasco da Gama Research Center / Vasco da Gama University School, Coimbra, 3020-210 Portugal; 5 Centre for Interdisciplinary Research in Animal Health (CIISA), Faculty of Veterinary Medicine, University of Lisbon, Lisbon, Portugal; 6 Pathology Laboratory, UEISPSA, National Institute for Agricultural and Veterinary Research (INIAV), I.P, Oeiras, 2780- 157 Portugal; 7 Animal and Veterinary Research Centre (CECAV), University of Trás-os-Montes and Alto Douro (UTAD), Vila Real, 5000- 801 Portugal; 8 Parasitology Laboratory, UEISPSA, National Institute for Agricultural and Veterinary Research (INIAV), I.P, Oeiras, 2780- 157 Portugal

**Keywords:** *Toxoplasma gondii*, New World non-human primate, Black-capped squirrel monkey, Microsatellites, Genotyping

## Abstract

New World monkeys are especially vulnerable to develop severe clinical manifestations and succumb to acute toxoplasmosis. This study aimed to describe the histopathological findings and genotypic characterization of the *Toxoplasma gondii* strain involved in a lethal case occurring in a zoo-housed black-capped squirrel monkey (*Saimiri boliviensis*) in Portugal. Cyst-like structures suggestive of Sarcocystidae parasites and acute injuries in liver and brain were observed by light microscopy examination. By immunohistochemistry, calprotectin, *T. gondii* antigen and Iba1 antigen had a positive signaling in lung, liver and brain tissues. *Toxoplasma gondii* B1, ITS1 and 529 repetitive element fragments amplifications together with the genotyping of 13 microsatellite markers confirmed a systemic *T. gondii* infection linked to a non-clonal type II strain. This description is consistent to the majority *T. gondii* strains circulating in Europe.

## Introduction

*Toxoplasma gondii* (Apicomplexa) represents one of the most successful protozoan parasites worldwide. Toxoplasmosis can become especially harmful to New World non-human primate (NWNHP) species, which act as their intermediate hosts (Dubey 2022) and can acquire the infection by horizontal or transplacental transmission (Juan-Sallés et al. 2011).

Squirrel monkeys are part of the NWNHP denomination, which includes various species of primates whose free-ranging populations are limited to tropical areas of the Americas (Dubey et al. 2021). Although information on the epidemiology of toxoplasmosis in free-living NWNHPs is scarce, several reports of acute outbreaks in captive squirrel monkeys evidence their high susceptibility to *T. gondii* infection; up to 100% morbidity rates with nonspecific signs and frequent lethality associated to sudden death have been described (Cunningham et al. 1992; Carme et al. 2009; Dubey et al. 2021). It has been proposed that ecological factors and behavioral habits have shaped their immune response capacity against this infection and may have influenced the high susceptibility of some primate colonies (Catão-Dias et al. 2013).

Genetically, *Toxoplasma gondii* is a highly diverse organism, with clonal (type I, II, III) and non-clonal lineages identified worldwide (Shwab et al. 2014). Genotypic features of *T. gondii* can be involved in its virulence, host range and tissue tropism. Wildlife in tropical and subtropical areas of America could be mainly exposed to non-canonical or highly diversified strains (Galal et al. 2019); whereas in Europe, type II strains are predominant among wild compartment and frequently detected in captive animals (Fernández-Escobar et al. 2022a).

The aim of the present study was to describe a fatal toxoplasmosis case in a *Saimiri boliviensis* from a zoo in Portugal and to provide genotypic data of the *T. gondii* strain detected.

## Materials and methods

### Case description

A four-year-old, male, captive black-capped squirrel monkey (*S. boliviensis*), born in Lagos Zoo (South Portugal), was found dead in its enclosure without signs of illness. No clinical cases or deaths had occurred in the population for over one year. However, three black-capped squirrel monkeys that died at the same zoo (two in November 2018 and one in November 2019) had presented with pleural and abdominal effusions and tested positive to toxoplasmosis by real-time Polymerase Chain Reaction (qPCR) in effusion fluid and tissue (tissue samples unspecified in the report). Additionally, six individuals were introduced into the group nearly three years ago, originating from an institution in France where *T. gondii* cases had been diagnosed. Two of those six individuals had already died, but the surviving animals remained in the same group as the subjet of this case. One of the remaining animals, a twelve-year-old female suffering from chronic blindness ultimately euthanized for welfare concerns, was diagnosed presumptively with chronic toxoplasmosis. Although no immunohistochemical nor molecular detection methods were applied, the presumptive diagnosis was made based on post-mortem findings of a non-suppurative meningoencephalitis, with gliosis and protozoan-like cystic forms (approx. 50 µm in diameter), with no cellular inflammatory reaction around them, in the molecular layer and white substance of the cerebellum.

### Macroscopic and histopathological examination

The necropsy of the present individual was carried out at the National Reference Laboratory for Animal Diseases (INIAV I.P., Portugal) for macroscopic lesion examination and for collecting tissue samples. For histopathological examination of liver, lung, spleen, kidney and encephalon, samples were fixed in 10% buffered formalin and embedded in paraffin using standard procedure. Five-µm thick sections were stained with hematoxylin and eosin (H&E) and examined using light microscopy (Olympus BX60 and Image Focus Alpha ® camera).

### Immunohistochemistry labelling

In order to investigate the localization of the parasite in relation to histological lesions, histological sections of the brain, lung and liver were attached to poly-l-lysine coated slides and left to dry at 37°C over-night. They were then immersed in a Tris-based solution (pH 9.0) and heated at 65°C for 20 min for deparaffinization, rehydration, and epitope retrieval (PT-Link System, Agilent Technologies). Samples were incubated overnight at 4°C with primary antibodies against Iba1 (1:1000 dilution, mAb NCNP24 clone, FUJIFILM Wako Chemicals U.S.A. Corporation), calprotectin (mAb 1:200, MAC387 antibody, Genetex) or *T. gondii* (1:2000 dilution, pAb, in-house) diluted in phosphate-buffered saline (PBS) in a humidified chamber. After washing, immunolabelling was performed using a ready-to-use kit ImmPRESS-AP Detection System (Vector Laboratories) incubating with the appropriate Polymer Anti-Mouse or Anti-Rabbit IgG Reagent for 30 min. After washing, the immunoreactivity was revealed using Vector Red substrate solution (Vector Laboratories) producing a red stain. Lastly, sections were rinsed in tap water and counter-stained with Mayer’s hematoxylin for 10 s, dehydrated through gradients of ethanol and xylene and coverslipped.

### Molecular detection

#### Conventional PCR from fresh tissue

Fresh brain tissue was homogenized with a syringe and 18G needle in a 15 ml Falcon tube; approximately 500 µl of tissue homogenate were withdrawn and resuspended in 200 µl PBS in a 1.5 ml tube. DNA was extracted from 200 µl tissue homogenate using the IndiMag Pathogen Kit (Indical Bioscience, Leipzig, Germany) on a fully automated KingFisher™ Flex Purification System (Thermo Scientific). *Toxoplasma gondii* DNA detection targeted the 35-fold repetitive glycerol-3-phosphate dehydrogenase (B1) gene (Hohlfeld et al. 1994). DNA from the type I RH strain (maintained in cell culture and kindly provided by *Instituto Nacional de Saúde Dr. Ricardo Jorge*) and nuclease-free water were used as positive and negative controls, respectively. PCR products were run on a 2% agarose gel stained with 2–3 µL of GreenSafe Premium in 1X TBE and the bands were visualized under UV light using a Gel Doc EZ Imager™ (Bio-Rad).

#### Quantitative PCR from paraffin block-embedded tissues

Paraffin-embedded samples were employed due to the availability of biological material for this technique and subsequent ones. DNA was extracted with the Maxwell® RSC DNA FFPE Kit (Promega, Madison, WI, USA) (block 1: 17975/22 brain; block 2: 17975/22 liver and lung). Five µm-thick slides from each block were used. In total, two replicates of two slides were subjected to deparaffinization and lysis following the manufacturer’s recommendations. DNA yielded was quantified by spectrophotometry (NanoPhotomer®, Implen GmbH, Munich, Germany).

*Toxoplasma gondii* DNA detection was attempted by single-tube nested PCR targeting the internal transcriber spacer 1 (ITS1) region by formerly described conditions (Hurtado et al. 2001) in a Veriti thermal cycler (Applied Biosystems, Waltham, Massachusetts, USA). DNA from the type I RH strain (10 ng/ml) and nuclease-free water were used as positive and negative controls, respectively. Amplicons of expected size (227 bp) were visualized by 1.5% (w/v) agarose gel electrophoresis in 1X TAE buffer, stained with Gel Red® Nucleid Acid Gel Stain (Biotium, Fremont, California, USA) and then observed under UV light. Amplicons were subjected to Sanger sequencing at the Center for Genomic Technologies of the Complutense University of Madrid (Spain). Obtained sequences were edited using BioEdit software, v.7.0.5.3. Alignments were carried out with the Clustal Omega Software (https://www.ebi.ac.uk/Tools/msa/clustalo/) and consensus sequences were compared with available sequences from the NCBI database through the BLAST tool (http://blast.ncbi.nlm.nih.gov/Blast.cgi).

Aiming at parasite quantification, samples resulting positive at the above nested PCR, were analyzed by duplex qPCR targeting the specific 529 repetitive element (RE) and a 189 bp internal amplification control (Homan et al. 2000; Slana et al. 2008) according to previously reported conditions (Fernández-Escobar et al., 2022b). DNA from the type I RH strain (10 ng/ml) and nuclease-free water were used as positive and negative controls, respectively. Reactions were performed in a 7500 FAST real-time PCR system (Applied Biosystems, Foster City, California, USA).

### Genotyping

Amplicons that reached less than 32 cycle threshold (Ct) values in duplex qPCR were subjected to genotyping by 15 microsatellite (MS) markers analyzed in a multiplex PCR assay (Ajzenberg et al. 2010). Following the indications by Joeres et al. (2023), the multiplex PCRs were carried out in a final volume of 25 µL, containing 12.5 µL 2X master mix, 2.5 µL Primer Mix (2 µM each primer), 9 µL DNase Free water and 1 µL DNA. The reaction included positive controls for the three clonal types (RH, Me49, and NED strains) at 5 ng/µl DNA concentration, along with nuclease-free water as a negative control. The cycling conditions were 95°C for 15 min; 94°C for 30 s, 61°C for 3 min and 72°C for 30 s (35 cycles); 60°C for 30 min, using a Veriti thermal cycler (Applied Biosystems, Waltham, Massachusetts, USA). Subsequently, PCR products were diluted (1:6) in Hi-Di formamide (Applied Biosystems) and then subjected to capillary electrophoresis with an ABI 3730 DNA Analyzer (Applied Biosystems, Waltham, Massachusetts, USA) at the Center for Genomic Technologies of the Complutense University of Madrid (Spain). Fragment size was measured using the bioinformatics software PeakScanner v.1.0 (ABI PRISM, Applied Biosystems, USA) and microsatellite typing results were determined following the guidelines by Joeres et al. (2023).

### Ancillary analyses

Samples consisting of intracardiac blood (hemoculture), a piece of lungs, liver, spleen and small intestine were aseptically collected and inoculated in culture media for bacteriological examination. Hemoculture was dispensed into Brain Heart Institution Broth (BD, USA) and the organs (except the small intestine) were macerated in physiological saline and inoculated onto Columbia Agar Base with 5% sheep blood and MacConkey Agar (Biomérieux, France), followed by incubation at 36 ± 1 ºC for 18–24 h. The intestinal contents were inoculated in Buffered Peptone Water (BD, USA) and followed ISO 6579-1:2017 (International Organization for Standardization 2017) for the search of *Salmonella* spp. Biochemical identification of colonies observed was performed through API32E (Biomérieux, France).

## Results

### Macroscopic and histopathological examination

**Fig. 1:**
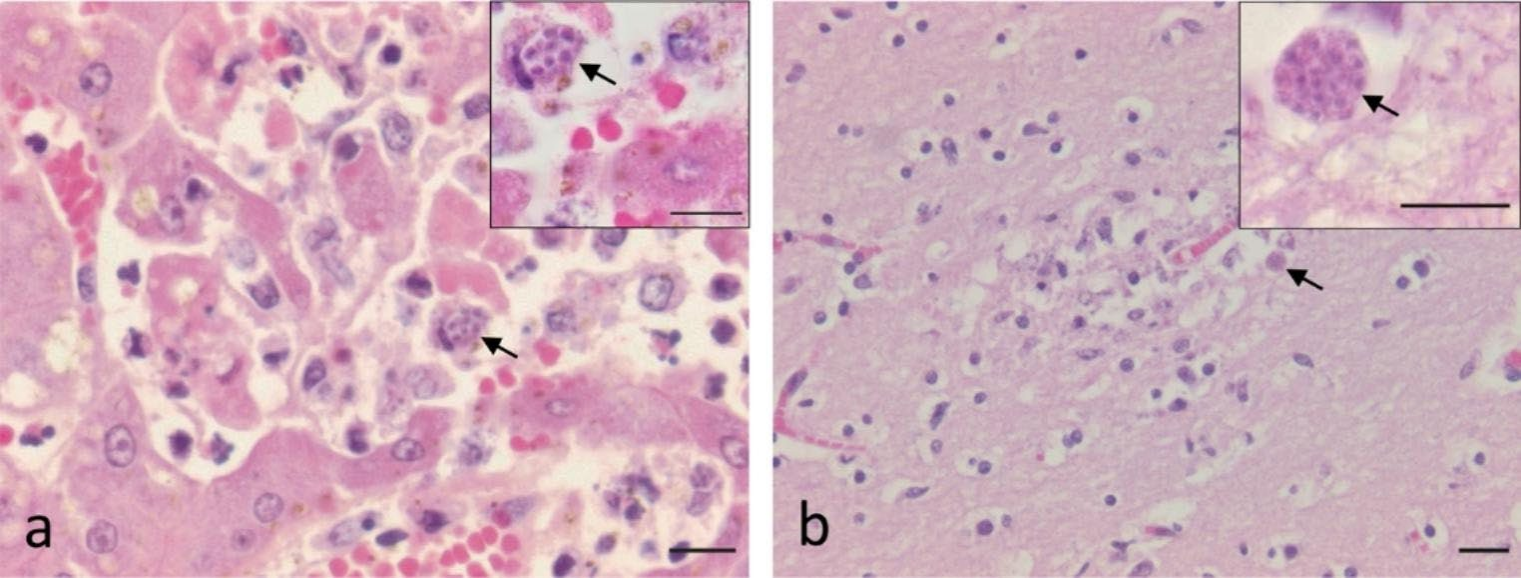
Histopathological features of the infected squirrel monkey. **a**). Liver: necrosis of hepatocytes with intralesional protozoan cystic structure (arrow); inset detail of the parasitic form (H&E, bars = 10 μm); **b**). Brain at the level of the thalamus: focus of gliosis in the white matter and intralesional protozoan cystic form (arrow), inset detail of the parasitic form (H&E, bar = 20 μm; inset bar = 10 μm)

Macroscopic lesions included 2.5 ml of intra-thoracic pleural effusion, congestion of the meninges and absence of gastric content. In the liver, a diffuse, severe acute hepatitis with multifocal necrosis of hepatocytes and diffuse, moderate infiltration of sinusoid capillaries was observed. Cystic parasitic forms suggestive of protozoa of the Apicomplexa group (Sarcocystidae family) were observed within Kupffer cells (Fig. 1a). In the brain, an intense congestion and scattered micro-hemorrhages were visualized. Foci of gliosis were also observed in the white matter of the thalamus and cerebellum. In one of the foci of gliosis located in the thalamus, a cystic parasitic form of a protozoan with a diameter of 8.6 μm was detected, compatible with Apicomplexan organism (Fig. 1b). Lung tissue was unsuitable for histopathological examination due to autolytic changes. No microscopic lesions were detected in the spleen or kidney samples.

### Immunohistochemistry labelling

**Fig. 2:**
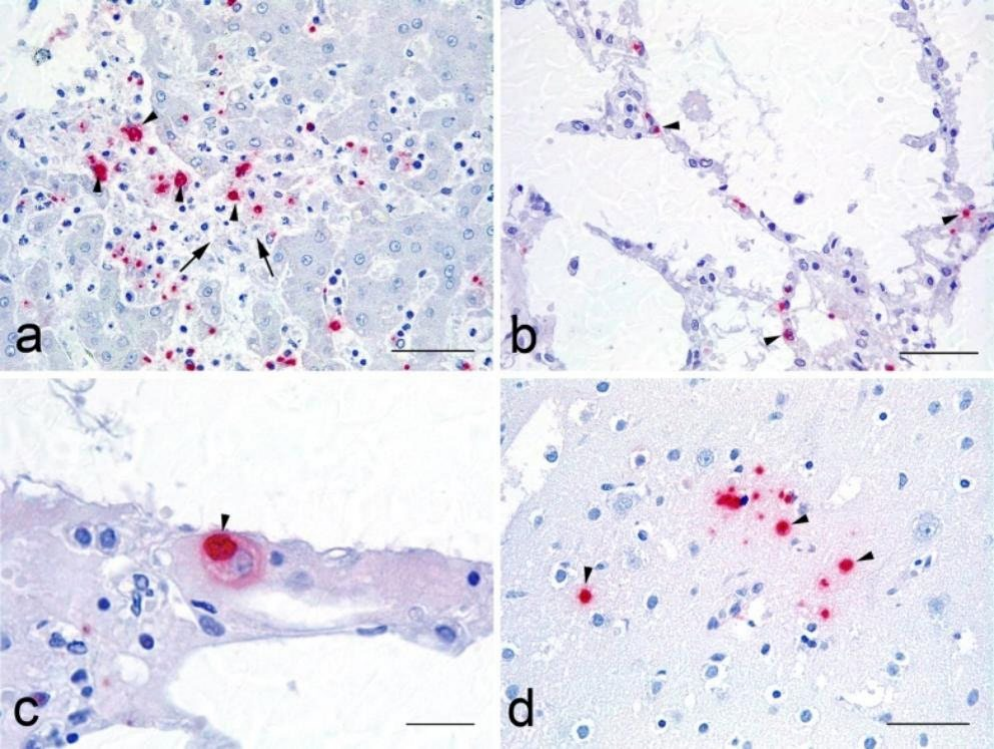
Immunohistochemical labelling of *Toxoplasma gondii* in tissue samples. **a**). Liver: intracellular labelling of *T. gondii* antigen inside mononuclear cells located at the central vein of the hepatic lobule but also infiltrating the adjacent parenchyma, close to a focus of necrosis (*T. gondii* IHC, bar = 50 μm); **b**). Lung: intracellular positive labelling of parasite antigen inside cells at the alveolar walls (*T. gondii* IHC, bar = 50 μm); **c**). Lung: high power magnification of positive labelling inside a mononuclear cell, morphologically consistent with a macrophage, at the alveolar wall (*T. gondii* IHC, bar = 20 μm); **d**). Brain: scattered labelling at the neuroparenchyma. No significant lesions could be found in the area (*T. gondii* IHC, bar = 50 μm)

The identification of parasite antigen showed evident differences between the liver, lung and brain (Fig. 2). The amount of positive labelling was higher in the liver, evident at low magnification, and related with the inflammatory infiltrate or necrotic foci within the parenchyma (Fig. 2a). In the lung, the positive signal was observed in the alveolar walls, frequently in relation to small vessels or capillaries (Fig. 2b). The antigen was seen in the wall of the vessels, possibly within endothelial cells, and also inside cells morphologically consistent with alveolar or intravascular macrophages (Fig. 2c). In the brain, the antigen presence was scarce, found within the neuroparenchyma and with no apparent relation to inflammatory lesions nor vessels (Fig. 2d).

**Fig. 3:**
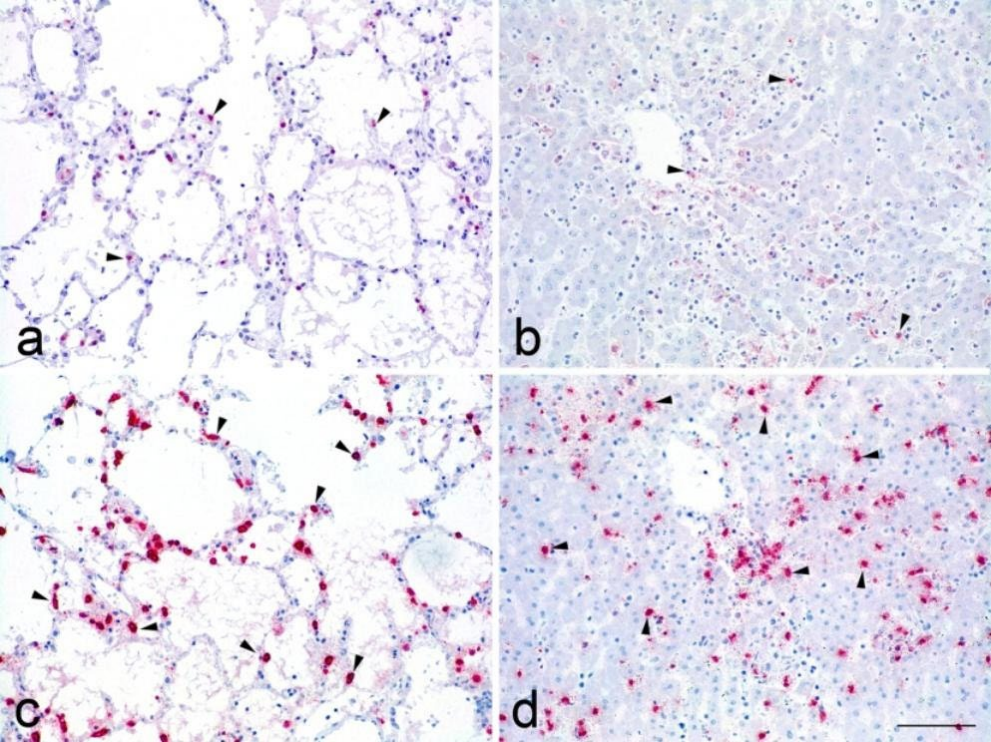
Immunohistochemical labelling of macrophages in tissue samples. **a**). Lung: few cells show positive labelling inside the alveolar walls (Iba1 IHC); **b**). Liver: similarly to **a**), the number of labelled cells is low (Iba1 IHC); **c**). Lung: serial section to **a**). The number of labelled cells is clearly higher than those labelled for Iba1 (calprotectin IHC); **d**). Liver: serial section to **b**), and, like **c**), the number of cells labelled by calprotectin is significantly higher than those labelled by Iba1 (calprotectin IHC). All pictures were taken at the same magnification (bar = 100 μm)

The labelling of Iba1 antigen was low to moderate in both the liver and the lung (Fig. 3a, b). In the brain, it was mainly located at the cerebral cortex. In none of the organs there was an evident relation between the location of the positive signal against Iba1 and *T. gondii* antigen. On the other hand, the labelling for calprotectin (i.e. MAC387 antibody) was abundant in both the lung and liver (Fig. 3c, d). Although no co-localization labelling was attempted in this study, there was a clear relation between the localization of calprotectin signal and that of the parasite antigen (Figs. 2a and 3d for the comparison in the liver).

### Molecular detection

A segment of the B1 gene (115 bp) was successfully amplified from fresh tissues of brain, liver and lung. The ITS1 (227 bp) partial sequence was also amplified from paraffin block-embedded tissues (block 1: brain; block 2: liver and lung). *Toxoplasma gondii* amplicons showed 100% identity to previous registered sequences in GenBank (e.g. accession numbers MH793505.1, MH793504.1, among others). Regarding the duplex qPCR, the number of *T. gondii* zoites were calculated by interpolating the Ct values obtained on a standard curve from 6 points generated by 10-fold dilutions of *T. gondii* DNA (10^5^ to 10^ − 1^ tachyzoites). qPCR reaction showed the following parameters: an average slope of − 3.3 and an R^2^ > 0.98. Parasite loads in brain (mean Ct = 32.8) and in the liver and lung (mean Ct = 28.2) yielded results of 1.14 and 28.1 zoites/mg of tissue, respectively.

### Genotyping

Attempt to amplify genotyping and fingerprinting MS markers is summarized in Table 1. Consensus profile is identified as a non-clonal type II strain.

**Table 1:** Microsatellite (MS) typing profile of *Toxoplasma gondii* infecting a squirrel monkey (*Saimiri boliviensis*) in Portugal

Reference strain / Specimen	Lineage	MS markers														
		N61	B18	M33	M48	TUB2	N83	XI.1	N82	TgM-A	W35	IV.1	B17	N60	M102	AA
RH	Type I	87	160	169	209	291	306	358	121	209	248	274	342	144	164	265
Me49	Type II	91	158	169	215	289	310	356	111	207	242	274	336	142	174	265
PRU	Type II	127	158	169	209	289	310	356	117	207	242	274	336	142	176	265
NED	Type III	91	160	165	209	289	312	356	111	205	242	278	336	147	190	267
Squirrel monkey*	Non-clonal Type II	89	158	169	223	289	308	-	117	207	242	274	336	140	174	-

* Consensus profile obtained after merging the allele identified in the two paraffin blocks examined (brain and liver and lung). -: No amplification

### Ancillary analyses

Only *Escherichia coli* was isolated by bacteriological examination from the hemoculture and tissue pool (liver, spleen and lung).

## Discussion

The present study is the first reported case of fatal acute toxoplasmosis in captive populations of squirrel monkeys in Portugal. Previously, high mortality rates have been described in the literature in outbreaks occurring on *S. boliviensis* (35.7%) and *S. sciureus* (ranging from 29.1 to 100%) (Cunningham et al. 1992; Bacciarini et al. 2001; Carme et al. 2009). At Lagos Zoo, both *S. boliviensis* and prosimian species (*Lemur catta*) with clinical signs have been previously diagnosed with toxoplasmosis and all subsequently died, but detailed parasite identification was not performed. As a result, toxoplasmosis has emerged as an important concern for the health and welfare of primates at the institution, warranting detailed diagnosis in order to implement preventative measures.

A higher parasite burden was found in liver and lung tissues (28.1 zoites/mg tissue) than in the brain (1.14 zoites/mg tissue). These findings are in agreement with previous reports of parasite loads ≈ 14 zoites/mg in the lung and 23 zoites/mg in the liver of two *S. sciureus* in Mexico (Cedillo-Peláez et al. 2011). Furthermore, the lung but also the heart (> 40 zoites/ng tissue) contained higher parasite loads than the brain, spleen, and kidney (Nishimura et al. 2019). Hence, heart, liver and lung tissues may be considered as suitable targets for molecular diagnosis during acute infections in squirrel monkeys. The amount of parasite’s antigen detected in the slides correlates with the molecular analyses, as the positive signal observed in the liver was evidently more abundant than that found in the brain. The lesions found in these three organs, especially the multifocal and severe hepatic necrosis, the higher abundance of parasite antigen in the liver than in the brain, and the absence of evident tissue cysts, suggest that the case corresponds to a subacute disease after a recent exposure, as previously reported in similar cases (Cunningham et al. 1992; Nishimura et al. 2019). This hypothesis is supported by the abundant expression of calprotectin, which is predominantly expressed in monocytes/macrophages recently recruited from the blood vessels and participating in active inflammation (Soulas et al. 2011). The distribution of the parasite antigen in the liver and particularly in the lung suggest that, as the time of death, the parasite was still disseminating through the systemic circulation and that the migration into the parenchyma of the organs was done through the infection of endothelial cells and/or by a Trojan horse strategy, both of them already demonstrated in different organs and cell populations (Randall and Hunter 2011). In this case, structures resembling parasitophorous vacuoles were found in the cytoplasm of cells morphologically consistent with macrophages. It is then tempting to hypothesize that, in the current case, these cells, possibly monocytes/macrophages, were acting as Trojan horse for the parasite.

The potential association between *T. gondii* genotype and pathogenicity in NWNHPs is still unknown, but genetic variations have been proposed as key factor (Dubey et al. 2021). The present case was caused by a non-clonal genetic variant related to archetypal type II strain, and this is in agreement with the predominant genotypes circulating in Europe (Fernández-Escobar et al. 2022a). A complete genotypic profile of 15 markers could not be obtained through MS analysis due to the absence of amplification for markers XI.1 and AA. A likely reason for this scenario is that the paraffin-embedded samples had a lower DNA concentration than what was estimated as optimal for amplifying the full panel of markers (Ajzenberg et al. 2010). Limited genetic typing data obtained by multilocus nested polymerase chain reaction – restriction fragment length polymorphism analysis (nPCR-RFLP) have revealed that acute toxoplasmosis cases in *Saimiri* spp. were associated with a type I-related strain in Mexico (by SAG3 locus analysis) (Cedillo-Peláez et al. 2011); with a type II-related strain in South Korea (by GRA6 locus analysis) (Oh et al. 2018); and with a type III-related strain in Israel (by SAG2 locus analysis) (Salant et al. 2009). The use of a greater number of genetic markers allowed the description of strains with a mixed pattern in Japan (ToxoDB #4; type I alleles in SAG1, SAG2, SAG3, BTUB, GRA6, c22-8, c29-2 and PK1 loci + type II alleles in L358 and Apico loci analysis, respectively) (Nishimura et al. 2019), and Argentina (ToxoDB #163; type I allele in c29-2 locus + type II allele in c22-8 locus + type III alleles in SAG2, BTUB, GRA6, SAG3, PK1, L358 and Apico loci) (Pardini et al. 2015). Regarding MS typing results, acute toxoplasmosis in *Saimiri* spp. has been associated with a type II strain in South Africa (isolates A10011a-d, analyzed by 15 MS markers) (Shwab et al. 2018), with a type II strain and a recombinant strain (Type I + III + non-canonical alleles) in French Guiana (both analyzed by 12 MS markers) (Carme et al. 2009). Including ours, all these strains have been lethal to *Saimiri* spp. individuals.

Type II strains are predominant among European animals and this fact may imply that zoo species housed in Europe are exposed to a limited number of strain genotypes if compared with their natural environments (Denk et al. 2022). Many outbreaks have been described in captive species, but those accompanied by genotypic data are scarce. In an Italian zoo, a possible type II strain determined by 8 PCR-RFLP markers was confirmed in one individual during a fatal outbreak in ring-tailed lemurs (*Lemur catta*) (Rocchigiani et al. 2022). We emphasize that isolation of circulating *T. gondii* strains among zoo species is a pending task, in order to obtain complete genotypic profiles and allow their phenotypic evaluation.

Finally, the source of infection could not be determined for this outbreak, but strict adherence to biosecurity measures is highly recommended in animal enclosures to reduce the risk of exposure to *T. gondii* between facilities, not only to endangered species but also for humans.

## Conclusion

This is the first description of a non-clonal type II strain with a lethal effect on *S. boliviensis* housed in Portugal, highlighting the potential threat of severe sickness and death to other captive NWNHPs due to the predominant lineage circulating in Europe.

## Data Availability

All data generated or analyzed during this study are included in this published article.
